# Domestic Medicine

**Published:** 1872-07

**Authors:** 


### INFORMATION FOR THE HOUSEHOLD.

PREPARED BY THE EDITOR.

TO CLEAN GOLD CHAINS.—Put the chain in a small glass bottle, with warm water or eau-de-cologne, a little camphorated chalk, and scrape in some soap. Cork the bottle and shake it for a minute violently. The friction against the glass polishes the gold.

---

CHEAP AND DURABLE WROUGHT NAILS.—Every farmer understands the usual method of making cut nails flexible by heating them; but if, instead of allowing them to cool in the open air, they are thrown when red hot into linseed oil, it will prevent their rusting almost as long as if they were galvanized.—*Boston Jour. Chem.*

---

ZINC WHITE FOR POLISHING SILVER, BRITANNIA, &c.—Those who have tested it say that the very best article for cleaning silver, and other metalic ware is zinc white, (No. 1 quality), used by painters. Rub it fine with a knife, sprinkle it on a soft flannel cloth, with which rub the ware, then brush off with tissue paper or any clean soft cloth.

---

POISONING FROM HAIR RESTORERS.—We have repeatedly warned our readers against the use of the so-called "hair restorers," of whatever name, as they all contain sugar of lead, which is very poisonous when absorbed by the vessels of the scalp. We have recently seen four well defined cases of lead-poisoning from the use of "Mrs. Allen's," "Hall's," and other hair preparations. All of the cases were complete paralysis of the extensor muscles of the fingers and hands.

---

A GOOD CEMENT.—The following has been tested for cementing wood, iron, leather, glass, paper, and almost all kinds of household materials. Best isinglass, half an ounce; rub it between the hands until it breaks down into a powder, put in a bottle and put as much common acetic acid to it as will just wet the mass through, stand the bottle in some boiling water and the paste will dissolve and be fit for use at once; it will be solid when cold, but is easily warmed up the same as before. Leave the cork out when warming or there is danger of bursting the bottle.

BLEACHING COMPOUND.--Stir 5 lbs. of chloride of lime into two pails of warm water; dissolve 10 lbs. of glauber salt, (sulphate soda), in 1 pail of water; also 4 lbs. of sal soda in 1 pail of water. The contents of the 4 pails can be poured together and kept in any suitable tight vessel. Such a quantity as the above ought to last a long time, as a dipper full of it would bleach a large quantity of linen or other goods. The materials are cheap, and the mixture is easily made.

---

TO PRESERVE FLOWERS.—It is often desirable to preserve flowers gathered by departed friends, or rendered sacred by having decorated their caskets. It can be easily and beautifully done by dipping the flowers when fresh, and perfectly free from moisture, into melted paraffine. The paraffine should not be heated any more than to just melt it. The flowers should be dipped in it and moved about until no bubbles of air appear about them. By this process they become hermetically sealed, and remain fresh and bright as when plucked from the garden.

---

POISON BY IVY.—Dr. J. D. Stewart, of Coila, N. Y., writes to the *Boston Journal of Chemistry*, that he has used the following remedy in poisoning by ivy, which at once arrests the tormenting, burning pain, and cures the worst cases in one or two days:

Bruise, slightly, a handful of white ash leaves; add new milk enough to cover; simmer ten minutes, and apply *as hot as can be borne*, three times a day.

—We reproduce the above article from the April number of the Bistoury, as the compositor then used the leaves of the wrong tree. He made us say white oak instead of white *ash*.

---

CRACKED WHEAT.—For a pint of the cracked grain, have two quarts of water boiling in a smooth iron pot, over a quick fire; stir in the wheat slowly; boil fast, and stir constantly for the first half hour of cooking, or until it begins to thicken; then lift from the quick fire and place where the wheat will cook slowly for an hour longer. Keep it closely covered, stir now and then, being careful not to allow it to burn on the bottom. When thus cooked, it is much sweeter and richer than when left to soak and simmer for hours. White wheat cooks easiest and is best. It may be eaten warm or cold, with milk and sugar. If eaten every evening, at tea, with nothing else, it will relieve obstinate constipation.

---

BOWEL COMPLAINTS.—Now that hot weather is upon us, children as well as adults, will be troubled with diarrhœa. The following will be found to be an excellent formula, for a cordial, to be kept always on hand for use in such diseases:

Take

Chalk mixture, 3½ oz;

Tincture Jamaica ginger, ½ oz.;

Laudanum, 1 drachm.

Mix and keep in a cool place. Shake the bottle before using. Dose, for an adult, one teaspoonful every hour until the pain and discharges cease. Children five years old, half a dose; younger ones in proportion.

---

IN CASE OF POISON.—Make your patient vomit by giving a tumbler of warm water with a teaspoonful of mustard in it, and then send for the doctor. If it be necessary to act without the doctor, and the poison is arsenic, give large quantities of milk and raw eggs, or flour and water. If the poison is an acid, give magnesia and water, or chalk and water, and plenty of warm water besides. If it is an alkali, like potash, give vinegar and water, lemon-juice, or some other safe acid. Always remember the emetic first. If it be laudanum, strong coffee is a good thing to give until the doctor comes. Keep the patient awake.

---

CANNED FRUITS.—The impression prevails among those who use the fruits freely which are put up in tin cans, that they are injured thereby, and this impression is in many cases correct.—We have long contended that all preserved fruits and vegetables should be stored in glass, and that no metal of any kind should be brought in contact with them. All fruits contain more or less of vegetable acids, and others that are highly corrosive are often formed by fermentation, and the metallic vessels are considerably acted upon. Tin cans are held together by solder, an alloy into which lead enters largely. This metal is easily corroded by vegetable acids, and poisonous salts are formed. Undoubtedly many persons are greatly injured by eating tomatoes, peaches, etc., which have been placed in tin cans, and we advise all our friends who contemplate putting up fruits the present summer, to use only glass jars for the purpose.

SHAVING FLUID.—Take of

| | |
|---|---|
| White hard soap (in shavings), | 1/4 lb. |
| Alcohol, . . . . | 1 pt. |
| Water, . . . . . | 1/4 lb. |

Perfume with bergamot, musk, or other perfume, as may be desired. Place the mixture in a strong bottle. Cork it close, set it in warm water for a short time, and occasionally agitate it briskly until solution is complete. Then pour off the clear portion from the dregs into clean bottles for use, and at once closely cork them. If the solution be not sufficiently transparent, a little alcohol should be added before decantation.

By simply rubbing a few drops on the skin, and applying the shaving brush, previously dipped in water, a good lather is produced.

---

POISONOUS COLORS.—Coal tar colors are frequently the cause of distressing symptoms in the human economy. Analine itself is a poison, and all colors that contain it in an unchanged state are consequently more or less toxic in their action. The agents employed in the preparation of analine colors, are in many instances very deleterious. Among these are the compounds of arsenic, zinc, tin, antimony, lead, together with hydrochloric and picric acids.

The common or inferior colors prepared from residues are especially dangerous, and are, on account of their cheapness, employed in coloring paper-hangings, wooden toys, matches, india-rubber articles, and confectionery. In the dyeing of woolen, and other tissues, the common analine colors are also extensively used, and sewing girls frequently suffer severely from the presence of arsenic and picric acid in their materials; their fingers become inflammed and dotted with small pimples upon a red ground; the same eruptions after awhile appear upon the face, the lips are of a dark violet color, and there is trembling of the hands and feet, accelerated pulse, and difficult respiration.

---

AUSTRALIAN CURE FOR SORE THROAT.—A correspondent of the *Queenslander* gives the following cure for sore throat: It cannot be too generally known that all forms of sore throat, whether simple, ulcerated, quinsy, diphtheria, scarlet fever, or otherwise, can be either totally cured or greatly alleviated by simply wearing a soft oil silk kerchief twice around the neck, high up and next the skin, especially if worn at night when the pain is first felt. Like Naaman, the Syrian, people will take any trouble but the right one, and fly to gargles, blisters, lotions, pills, etc., and keep at them for a month at a time; but an old silk square—why, it's too absurd, and so they hug their sore throat, and wonder why it don't get better. Not only does the silk cure the sore throat, but it prevents a recurrence of it. I was formerly a martyr to quinsy and ulcerated sore throat, and used to have a whole month of it regularly every winter, and in spite, too, of all the usual battery of pills, gargles, etc., it run its course till I tried the silk; the sore throat then took the hint and left me alone ever since as a bad customer. I invariably killed it within an hour of any attempt it makes upon me; an old sore throat will take a day to cure. Mind, I do not pretend to say that the silk will cure fever or any other symptom or complication that may accompany sore throat, but this I do say, that it will cure and remove all pain and difficulty of swallowing in the throat without the aid of any local remedy, or it will do it in spite of them, if you do apply them and it both, but, without it, cure only comes by nature, not physic, as far as the sore throat goes. Other remedies do neither good nor harm, except as they keep you from trying the infallible silk.

---

NOISE IN THE SICK ROOM.—We have picked up the following stray article without knowing from whence it came. The suggestion is such a good one, and one that is so much needed in every sick room where a stove is used, that we give it as we found it, heartily recommending it:—

"You know what a racket is caused even by the most careful hand in supplying coal to a grate or stove, and how, when the performance is undertaken by a servant, it becomes almost distracting. If you don't remember, take notice the first time you are ill, or you have a dear patient in your care, or the baby is in a quiet slumber. Let some one bring on the coal scuttle or shovel, and revive your recollection.—Well, the remedy we suggest is to put the coals in little paper bags, each holding about a shovelful. These can be laid quietly on the fire, and, as the paper ignites, the coals will softly settle in place. You may fill a coal scuttle or box with such parcels ready for use. For a sick room, a nursery at night, or even the library, the plan is admirable. Just try it. Besides, it is so cleanly. If you don't choose to provide yourself with paper bags, you can wrap the coals in pieces of newspaper at your leisure, and have them ready for use when occasion requires.

ONIONS.—Dr. John B. Wolf, of Washington, made the following communication to the Farmer's Club, of New York, recently: On ship-board at New Orleans, in 1849, in charge of one hundred marines, with cholera among them, I observed that those who ate freely of onions, supposing them to be healthy, were attacked certainly and fatally. Onions and salt cured the bite of a rattlesnake on my son, and are considered specific in all snake-bites. I have found four separate witnesses of phenomena connected with small-pox and fever:

1. Onions in rooms with small-pox rot rapidly.
2. Blisters rise in them.
3. They retain and communicate the virus many weeks after the epidemic has subsided.
4. Applied to the feet of fever patients they rapidly turn black.
5. They prevent the spread of small-pox in thickly populated tenements, by absorbing the virus.
6. A man with hydrophobia, in his frenzy, ate voraciously of onions and recovered.

From these facts may be deduced:

1. That onions should not be eaten when there is a prevailing epidemic.
2. That onions sliced and frequently changed are good disinfectants.
3. That experiments should be made to test the extent of their usefulness.

---

HOW SUMMER SUITS SHOULD BE WASHED.—Summer suits are nearly all made of white or buff linen, pique, cambric or muslin, and the art of preserving the new appearance after washing is a matter of the greatest importance. Common washerwomen spoil everything with soda, and nothing is more frequent than to see the delicate tints of lawns and percales turned into dark blotches and muddy streaks by the ignorance and vandalism of a laundress. It is worth while for ladies to pay attention to this, and insist upon having their summer dresses washed according to the directions which they should be prepared to give their laundresses themselves. In the first place, the water should be tepid, the soap should not be allowed to touch the fabric; it should be washed and rinsed quick, turned upon the wrong side, and hung in the shade to dry, and when starched, (in thin boiled, but not boiling starch), should be folded in sheets or towels, and ironed upon the wrong side, as soon as possible. But linen should be washed in water in which hay has been boiled, or a quart-bag of bran. This last will be found to answer for starch as well, and is excellent for print dresses of all kinds, but a handful of salt is very useful also to set the colors of light cambrics and dotted lawns; and a little beef's gall will not only set, but brighten yellow and purple tints, and has a good effect upon green.

---

PAPERING ROOMS.—Don't try to paper with a carpet down. Make paste, cut bordering, and the paper, the day before. If the wall has been whitewashed, it must be washed in vinegar to neutralize the alkali in the lime. If papered before and you wish the paper removed, sop with water and it will peel off.

If convenient, provide a long board, wide as the paper, though a table or two will do. The paper must be measured, placed right side down on the board; then with a brush proceed to lay on the paste, not too thickly, but over every part, and be careful that the edges receive their share. When completed, double within three inches of the top, the paste sides being together; carry to the wall, mount your chair, and stick your three inches pasted paper on the wall at the top. That holds it; now strip down the other, and see that it fits just right; if not, peel down, make right, then press to the wall from the center right and left. Leave no air under, or when warm it will expand, bursting the paper.

Of course, the paper must be matched; it will not do to measure by line unless the walls are perfectly plumb. Small figures make less waste, and make a small room look larger.—Stripes make a low room higher, and if there are no figures between, or in the stripe to match, there is no waste, and no trouble in putting on. If a narrow border is the style, let it be bright, if the paper be neutral, but if that be bright, the border had better be dark and neutral.

If the paste be made too thick, the paper will be apt to crack and peal off; if too thin, it will saturate the paper too quickly and make it tender in putting on. A counter duster, (Brussels brush), is nice to brush the paper to the wall.—White, clean cloths will do, but it will not do to rub the paper with this; being damp, the paint or color rubs off the paper. The tables must be dried each time after pasting, for the same reason. Paste under paper must not freeze, neither dry too quickly. If whitewashing is done after papering, tack double strips of newspapers wider than the border all around the room.—*Boston Jour. Chemistry.*

